# α-Synuclein induces prodromal symptoms of Parkinson’s disease via activating TLR2/MyD88/NF-κB pathway in Schwann cells of vagus nerve in a rat model

**DOI:** 10.1186/s12974-023-02720-1

**Published:** 2023-02-14

**Authors:** Yue Cheng, Qing Tong, Yongsheng Yuan, Xinna Song, Wenwen Jiang, Yueping Wang, Wenjie Li, Yangxia Li, Kezhong Zhang

**Affiliations:** 1 Department of Neurology, The First Affiliated Hospital of Nanjing Medical University, Nanjing, 210029 China; 2 Jiangsu Key Laboratory of Neurodegeneration, Department of Pharmacology, Nanjing Medical University, Nanjing, 211116 China

**Keywords:** Parkinson’s disease, Prodromal phase, Autonomic dysfunction, Schwann cells, Neuroinflammation

## Abstract

**Background:**

Increasing evidence suggests that patients with Parkinson's disease (PD) present with peripheral autonomic dysfunction (AutD) that even precedes motor deficits, through which α-synuclein can spread to the central nervous system. However, the pathological mechanisms underlying AutD in prodromal PD remain unclear. Here, we investigated the role of α-synuclein and its interplay with the activation of Schwann cells (SCs) of the vagus nerve in AutD.

**Methods:**

Rats were subjected to injection with adeno-associated viruses containing the human mutated A53T gene (AAV-A53T) or an empty vector into the left cervical vagus nerve and evaluated for gastrointestinal symptoms, locomotor functions, intestinal blood flow, and nerve electrophysiology. Further, we examined the impact of α-synucleinopathy on vagus nerves, SCs, and central nervous system neurons using electron microscopy, immunofluorescence, immunohistochemistry, and western blot. Finally, the role of Toll-like receptor 2 (TLR2) in regulating the neuroinflammation in the vagus nerve via MyD88 and NF-κB pathway was determined using genetic knockdown.

**Results:**

We found that rats injected with AAV-A53T in the vagus nerve exhibited prominent signs of AutD, preceding the onset of motor deficits and central dopaminergic abnormalities by at least 3 months, which could serve as a model for prodromal PD. In addition, reduced intestinal blood flow and decreased nerve conduction velocity were identified in AAV-A53T-injected rats, accompanied by disrupted myelin sheaths and swollen SCs in the vagus nerve. Furthermore, our data demonstrated that p-α-synuclein was deposited in SCs but not in axons, activating the TLR2/MyD88/NF-κB signaling pathway and leading to neuroinflammatory responses. In contrast, silencing the TLR2 gene not only reduced inflammatory cytokine expression but also ameliorated vagal demyelination and secondary axonal loss, consequently improving autonomic function in rats.

**Conclusions:**

These observations suggest that overexpression of α-synuclein in the vagus nerve can induce symptoms of AutD in prodromal PD, and provide support for a deeper understanding of the pathological mechanisms underlying AutD and the emergence of effective therapeutic strategies for PD.

**Supplementary Information:**

The online version contains supplementary material available at 10.1186/s12974-023-02720-1.

## Introduction

Parkinson's disease (PD), in terms of prevalence, is one of the most rapidly growing neurodegenerative diseases worldwide due to a range of factors such as environmental influences and population aging [1]. Although PD is defined primarily as the occurrence of motor features, mounting evidence demonstrates that non-motor symptoms including sleep disturbances, cognitive impairments, and autonomic dysfunction (AutD) considerably contribute to the overall disease burden [2, 3]. Notably, several studies have demonstrated that AutD can increase the risk of developing PD and is associated with faster disease progression as well as shorter survival in patients with PD [4–7].

Known as a crucial clinical characteristic of PD, AutD covers a range spectrum of gastrointestinal dysfunction, cardiovascular impairment, urinary tract abnormalities, and sexual disturbances [8]. Recently, a large multicenter PD cohort study was conducted in Chinese patients, in which non-motor symptoms were systematically evaluated and revealed the prevalence of AutD to be up to 90% [9]. In addition, the overall picture is gastrointestinal dysfunction, typically featured by constipation, is the most common and more disabling AutD [10]. Furthermore, experimental and observational research has provided solid evidence for constipation as a diagnostic marker for prodromal PD, which can precede PD motor diagnosis by 15 years or more, and subsequently engage in the development of PD throughout the disease duration [10, 11]. However, although crucial for early diagnosis and initiating management strategies in the prodromal phase of PD, the pathophysiological basis of AutD and its potential role in disease development remains to be firmly established. Also, an appropriate animal model for the AutD in prodromal PD is yet to be addressed.

Pathologically, PD is characterized by the accumulation of α-synuclein in dopaminergic neurons of the substantia nigra in form of Lewy bodies and Lewy neurites, the primary component of which is Ser129 phosphorylated α-synuclein (p-α-synuclein). However, evidence for abnormal aggregation of α-synuclein outside the brain, including nerves and tissues of multiple peripheral systems, is growing [12]. Several autopsies and biopsies studies have found that p-α-synuclein deposits in the autonomic nerves of cardiovascular, gastrointestinal, salivary glands, and skin, partially occur in prodromal or early PD [13–17]; although these findings have yet to elucidate the specific locations of p-α-synuclein expression in neural tissue. Indeed, findings from our previous studies evaluating the sural nerve of patients with PD and the sciatic nerve of animal models identified the positive expression of p-α-synuclein was up to 100% and revealed for the first time that p-α-synuclein predominantly located in the myelin Schwann cells (SCs) [18–20].

Over the past decades, converging evidence has highlighted the spread of α-synuclein from the peripheral to the central nervous system (CNS), with the vagus nerve serving as a “highway” through which pathology could be transmitted to the brainstem [21]. The original Braak staging of brain pathology for PD suggests that the α-synucleinopathy initiates in the dorsal motor nucleus of the vagus (DMNV) and the anterior olfactory nucleus [22]. Accordingly, numerous studies have investigated the association between the vagus nerve and the risk or therapy of PD. In two large cohort studies, complete truncal vagotomy was shown to be engaged in a substantially decreased risk of developing PD [23, 24]. Interestingly, observations from clinical and experimental studies suggest that stimulation of the vagus nerve could improve gastrointestinal symptoms and gait impairment in PD patients, as well as motor deficits and dopaminergic neurotoxicity induced by 6-OHDA, which is attributable to the inhibition of neuroinflammatory [25–29]. However, it remains to be determined how the vagus nerve participates in the AutD in PD.

Hence, we hypothesized that p-α-synuclein deposited in SCs of the vagus nerve in prodromal PD and induced the pathological progression of AutD. In the current study, we constructed a prodromal PD model by injecting adeno-associated viruses (AAVs) containing a human mutated A53T form of the α-synuclein gene into the left cervical vagus nerve, which exhibited gastrointestinal AutD and disruption of neural integrity 3 months prior to the motor symptoms and loss of dopaminergic neurons. Moreover, the vagus nerve dysfunction was further verified by reduced intestinal blood flow, decreased nerve conduction velocity, and ultrastructural abnormalities including disruption of myelin sheaths and degeneration of SCs. Additionally, our results demonstrated that p-α-synuclein was deposited in SCs of the vagus nerve and activated the Toll-like receptor 2 (TLR2)/MyD88/NF-κB signaling pathway, leading to neuroinflammatory responses, while silencing the expression of TLR2 reduced nerve structure damage and improved functional outcomes. In aggregate, these findings uncover an unrecognized pathway that p-α-synuclein deposits in vagal SCs and induces a neuroinflammatory response involved in the development of AutD, providing a novel strategy and molecular target for the diagnosis and treatment of AutD in prodromal PD.

## Materials and methods

### Animals

Male Sprague Dawley rats (2-month-old, weight 240–260 g) were purchased from Viton Lever and housed in a temperature and humidity-controlled room at the Animal Core Facility of Nanjing Medical University, with a 12 h light/dark period and free access to water and food. All animal experiments were performed in accordance with animal ethics protocols and approved by the Institutional Animal Care and Use Committee of the Nanjing Medical University Experimental Animal Department.

### Human tissue samples

Sural nerve tissue samples were recruited from the PD patient database and the pathology database of the First Affiliated Hospital of Nanjing Medical University. A total of 10 patients with PD and 10 controls matched for age, gender, and free of neurodegenerative diseases were enrolled. The study was approved by the Ethics Committee of the First Affiliated Hospital of Nanjing Medical University. All subjects were fully informed of the aims of the study and provided written informed consent prior to participation.

### Adeno-associated virus

SD rats were injected with AAVs containing a human mutated A53T form of the α-synuclein gene (AAV2/6-CMV bGlobin-human-A53Talpha-synuclein-EGFP-3FLAG-WPRE-hGH polyA, 1 × 10^13^ GC/mL) or with an empty vector (AAV2/6-CMV bGlobin-empty vector-EGFP-3FLAG-WPRE-hGH polyA, 1.42 × 10^13^ GC/mL) (Genechem Co., Shanghai, China). In addition, AAVs carrying TLR2 sagRNA were employed to silence TLR2 gene expression (AAV2/6-CMV-NLS-SaCas9-NLS-3xHA-bGHpA-U6-sgRNA, 1.23 × 10^13^ GC/mL) (Genechem Co., Shanghai, China).

### Cervical vagus nerve injections

To mimic the pathology in the prodromal phase of PD, we utilized the vagus nerve injection of AAV-A53T with reference to a previous study [30]. The rats were anesthetized with an intraperitoneal injection of pentobarbital sodium (40 mg/kg), positioned supine on a dissecting plate, and maintained at 37 °C with a heating pad. The hairs around the left neck were removed and the skin was disinfected with 70% alcohol, followed by making an incision near the middle of the neck, separating the tissue layer by layer, and exposing the left carotid sheath. The vagus nerve was isolated for approximately 1 cm and then gently suspended with sterile sutures to maintain a certain tension that would facilitate subsequent nerve injections and avoid viral contamination of the surrounding tissue. Next, the vagus sheath was pierced with a Hamilton syringe (0.16 mm tip diameter) and a 34G needle (0.06 mm inner diameter) with a depth of 0.2–0.4 mm in the direction of the nerve, and 2 µL of vehicle solution was injected at a rate of 0.5 µL/min. After the injection, the syringe was left for an extra 3–4 min and then slowly retracted and the skin was sutured. Lastly, alcohol disinfection was performed again and the rats were kept warm until waking up. The animals were monitored postoperatively and administered carprofen (5 mg/kg) subcutaneously for 3 days as an analgesic treatment. Rats were randomly divided into AAV-EV (*n* = 12), AAV-A53T (*n* = 12), AAV-Cas9-TLR2 (*n* = 12), and AAV-A53T + AAV-Cas9-TLR2 (*n* = 12) groups. In addition, rats without any treatment were employed as the control group (*n* = 12) to minimize the interference of surgical operations- and viral vector-induced vagus nerve damage on experimental results.

### Behavioral tests

To evaluate vagal injection of AAV-A53T-induced behavioral deficits, all rats were subjected to gastrointestinal function assessment 1 month after injection, which was performed once a week till the development of significant gastrointestinal dysfunction. Furthermore, all rats were assessed by the rotarod test and open field test for parkinsonian locomotor dysfunction. The experimenter was blinded to treatment group classification for all behavioral studies.

### Colonic transit time

Colonic transit time was assessed as described before [31]. Briefly, rats were fasted for 24 h and anesthetized with isoflurane. Steel beads (5 mm in diameter) were then slowly pushed into the colon located at a distance of 5 cm from the anal verge via a rounded glass rod. The time taken to excrete the beads was recorded by an experimenter with a stopwatch and considered as an estimate of the colonic transit time.

### Stool collection

After fasting for 24 h, rats were housed in a clean cage individually with free access to food and water. The stools were collected immediately after excretion and placed in sealed tubes, as well as the number and shape of fecal pellets were recorded. Collection time was from 8:00 am to 20:00 for a total of 12 h. Finally, the stools were counted and weighed (total weight), followed by drying in a 56 °C oven for 48 h and weighing again to obtain the dry weight. The stool water content was the ratio of the difference between the total weight and dry weight to the total weight.

### Rotarod test

To verify whether the rat model would eventually develop PD, the rotarod test was conducted to evaluate motor coordination abilities, which is the core symptom of PD. Three days prior to the test, rats were trained with a rotating rod behavioral apparatus (Jiliang Pharmaceutical Engineering Co., Shanghai, China) at a slow rotational speed (5 rpm) for 10 min each time. The time that rats kept on the rotating bar was recorded at a rotational speed of 25 rpm during the formal test. Rats without dropping for more than 5 min were recorded as 300 s.

### Open field test

The open field test was conducted to assess behavioral activity and autonomous locomotion. In this test, rats were individually placed in the central area of a box (90.2 × 90.2 × 61 cm) with a black background, and then the trajectory was monitored and recorded for 5 min. The total distance of autonomous locomotion and the average velocity were analyzed using Openfield software (CleverSys Inc., VA, USA).

### Intestinal blood flow assessment

Intestinal blood flow was examined using a laser Doppler flowmetry as previously described [32]. In brief, rats were anesthetized with isoflurane and placed on a thermostatic table. After making an abdominal midline incision of approximately 2 cm, the mesentery near the cecum segment was gently spread into a fan shape and placed on a gel containing saline. Next, a low-power laser beam was steered onto the exposed intestine using a computer-controlled optical scanner with the scanner head placed parallel to the exposed intestine at a distance of approximately 20 cm. Subsequently, color-coded images indicating specific relative perfusion levels were shown on a video monitor. Blood flow values were recorded and calculated in perfusion units (PU) utilizing the Moor FLPIR V40 program (Gene and I Scientific Co., Beijing, China).

### Vagus nerve electrophysiology

Nerve conduction investigations were performed using an electromyography system (Haishen Medical Electronic Instrument Co., Shanghai, China) referring to the previous study [33]. Briefly, the rat was anesthetized with isoflurane and the cervical vagus nerve was exposed for approximately 2 cm, then a stimulation electrode was placed at the proximal part while two recording electrodes were placed spaced 1 cm at the distal part of the vagus nerve, with the ground electrode located around the stimulating and recording electrodes for ensuring that the impedance was less than 1 Ω. The electric current for stimulating the nerve was increased gradually until it attained a supramaximal level (0.1 ms, 1 Hz, 9.4 mA). Finally, the vagus nerve conduction velocity was calculated from the difference in latency of the nerve compound action potential between the two recording electrodes, with the waveform and amplitude being recorded.

Following electrophysiological testing, rats were euthanized, with vagus nerves and brain tissues being extracted and processed for transmission electron microscopy, immunofluorescence, immunohistochemistry, and immunoblotting.

### Transmission electron microscopy (TEM)

Transmission electron microscopic (TEM) studies were conducted as described previously [34]. In brief, vagus nerve sections were deposited on Formvar-coated 400 mesh copper grids, fixed with 2.5% glutaraldehyde, negative stained with 2% uranyl acetate, and visualized by a JEM 1400 TEM (JEOL, Tokyo, Japan).

### Immunofluorescence

For immunofluorescence analysis, paraffin-embedded rat vagus nerve section (5 μm) were dewaxed and rehydrated, then subjected to antigen retrieval using citrate buffer (pH 9) at 95 °C for 45 min. Next, sections were incubated with a blocking solution containing 0.3% Triton X-100 for permeabilization and 3% BSA for 20 min at room temperature, and then incubated with the following primary antibodies at 4 °C overnight: Anti-alpha-synuclein (Abcam ab280377, 1:500), Anti-phospho S129 alpha-synuclein (Abcam ab51253, 1:500), Anti-S100 beta (Abcam ab52642, 1:500), Anti-S100 beta (Proteintech 66616-1-Ig, 1:500), Anti-Neurofilament heavy polypeptide (Abcam ab207176, 1:200), Anti-MJF-14 (Abcam ab209538, 1:500), Anti-TLR2 (Abcam ab209216, 1:500), Anti- interleukin-1 beta (IL-1β) (Santa cruz sc-12742, 1:50). After washing 3 times in PBS, nerve sections were incubated with corresponding secondary antibodies at 1:2000 (Abcam, Alexa Fluor conjugates) for 1 h at room temperature. DAPI (Sigma-Aldrich) was used for staining nuclei. Images were acquired with a confocal laser scanning microscopy (Zeiss LSM700, Oberkochen, Germany).

### Immunohistochemistry

Immunohistochemistry was performed on 20 μm thick serial frozen rat brain sections and 5 μm thick paraffin-embedded human sural nerve sections. Sections were treated with 0.3% H_2_O_2_ for 15 min, then washed 3 × 5 min with PBS and incubated with 0.3% TritonX-100 for an additional 15 min, followed by washing 3 times in PBS and blocking with 5% goat serum for 1 h at room temperature. Primary antibodies were then diluted in a blocking solution and incubated overnight at 4 °C. The following primary antibodies were used: Anti-TH (Proteintech 25859-1-AP, 1:500), Anti-ChAT (ABclonal A13244, 1:500); Anti-TLR2 (ABclonal A11225, 1:500). After 3 washes for 5 min, secondary antibodies (Proteintech goat anti-Mouse SA00001-1, 1:1000, Proteintech goat anti-Rabbit SA00001-2, 1:1000) diluted in blocking buffer were incubated for 2 h at room temperature. All stereological analyses were performed using the 200× objective of an Olympus BX52 microscope (Olympus America Inc., Melville, NY, USA). The number of Choline acetyltransferase (ChAT)-immunoreactive cells in DMNV was calculated by Image J software (National Institutes of Health, Bethesda, Maryland, USA), and the number of Tyrosine hydroxylase (TH)-immunoreactive cells in the substantia nigra pars compacta (SNc) of the midbrain was assessed with an optical fractionator (Stereo Investigator 7, MBF bioscience, Williston, VT, USA). For the ChAT-positive neurons, 3 brain sections of the DMNV were counted in each rat using Image J and then averaged for 6 rats per group. The TH-positive neurons in the SNc of the rat midbrain were counted as previously described [35]. The regions of the SNc were outlined at low magnification (40×), and the counting frame size was 50 μm × 50 μm while the sampling grid size was 100 μm × 100 μm. Within 1 counting frame, positive cells counted must show the cell body. The sampling scheme was designed to have a coefficient of error < 10% to get reliable results.

### Western blot

Vagus nerve tissues were lysed in RIPA buffer (Research products R26200-250.0) supplemented with protease inhibitor cocktail (Roche, USA) using an ultrasonic cell disruption system (Sonics & Materials Inc., USA), and then centrifuged at 16,000*g* for 15 min at 4 °C. The concentrations of protein samples were quantified by BCA assay (Keygen Biotech, Nanjing, China) referring to the manufacturer's instructions, of which 30 μg was boiled in SDS loading buffer and then loaded in 8–12% acrylamide gels. After electrophoresis, the samples were transferred to a polyvinylidene difluoride (PVDF) membrane (Millipore, Billerica, MA, USA) and blocked with TBS containing 0.1% Tween 20 (TBST) and 5% non-fat milk at room temperature for 2 h. The following primary antibodies were incubated at 4 °C overnight: Anti-alpha-synuclein (Abcam ab280377, 1:2000), Anti-phospho S129 alpha-synuclein (WAKO 015-25191, 1:2000), Anti-TLR2 (ABclonal A11225, 1:2000), Anti-MYD88 (Cellsingnsal 4283 s, 1:1000), Anti-phospho-p65 (Cellsingnsal 3033S, 1:1000), Anti-IL-1β (Santa cruz sc-12742, 1:200). Subsequently, the membranes were washed 3 times in TBST and incubated with the corresponding HRP-conjugated secondary antibodies (Proteintech goat anti-Mouse SA00001-1, 1:5000, Proteintech goat anti-Rabbit SA00001-2, 1:5000) diluted in TBST at room temperature for 1 h. Western blot bands were acquired using the ECL assay kit and analyzed with the ImageQuant™ LAS 4000 imaging system (GE Healthcare, USA) equipped with the Image J software (National Institutes of Health, NIH).

### Statistical analysis

Statistical analysis was performed using GraphPad Prism 8.0 software. Student’s *t *test was used for two group comparisons. One-way analysis of variance (ANOVA) or two-way ANOVA followed by Bonferroni’s post hoc test was applied for comparison among three or more groups. Data are presented as mean ± SEM. Differences with *P* < 0.05 were considered to be statistically significant.

## Results

### Selective induction of α-synucleinopathy in the vagus nerve triggers gastrointestinal symptoms and precedes motor deficits and CNS pathology

**Fig.1:**
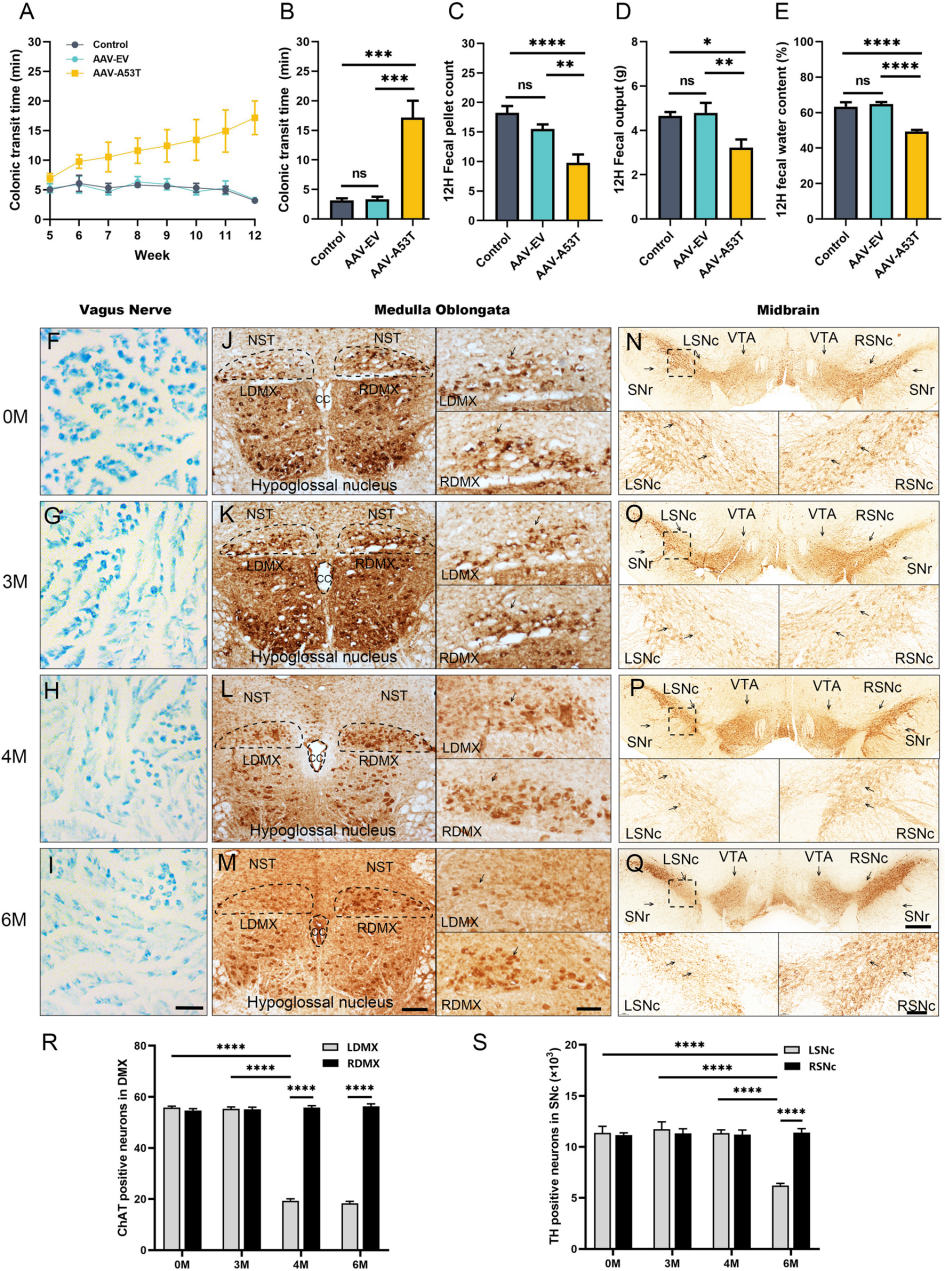
Vagal injection of AAV-A53T induces gastrointestinal dysfunction and progressive neuron degeneration in the CNS. SD rats were subjected to vagal injection with AAVs containing empty vectors (AAV-EV) or A53T (AAV-A53T) and then evaluated with gastrointestinal autonomic function 4 weeks later. The control group was male SD rats without any treatment. **A** Colonic transit time changes over time. After 3 months of injection, colonic transit time (**B**), 12 h fecal pellet count (**C**) and total weight (**D**), and fecal water content (**E**) in rats. Representative images of Luxol fast blue staining of myelin in the left vagus nerve (**F–I**), immunohistochemical staining of ChAT in the medulla oblongata (**J–M**), and TH in the substantia nigra (**N–Q**) at 0, 3, 4, and 6 months after injection of AAV-A53T, respectively. Scale bars = 10 μm in **F–I,** 200 μm for the left column and 100 μm for the right column in **J–M,** 500 μm for the top row and 100 μm for the bottom row in **N–Q**. The number of ChAT positive neurons in the DMX (**R**) and TH positive neurons in the SNc (**S**). **A–E**: *n* = 12 per group; **F–S**: *n* = 6 per group. Data were presented as mean ± SEM. Statistical significance was analyzed using one-way ANOVA in **A–E** and two-way ANOVA in **R** and** S**, followed by Bonferroni’s multiple comparison test. *P < 0.05, **P < 0.01, ***P < 0.001, ****P < 0.0001, ns, not significant; CNS, central nervous system; ChAT, choline acetyltransferase; TH, tyrosine hydroxylase; NST, nucleus of solitary tract; cc, central canal; L/RDMX, left/right dorsal motor nucleus of vagus nerve; L/RSNc, left/right substantia nigra pars compacta; VTA, ventral tegmental area; SNr, substantia nigra pars reticulata

To investigate whether α-synucleinopathy confined to the vagus nerve reproduces AutD in prodromal PD, we injected AAVs containing the human mutated A53T form of the α-synuclein gene or with an empty vector into the left cervical vagus nerve of SD rats and performed gastrointestinal autonomic function assessments weekly after 1 month of injection. Interestingly, 3 months later, colonic transit time assays showed that AAV-A53T-injected rats experienced a significant increase in the time to expel the steel beads (Fig. 1A, B; Additional file 1), indicating a deficit in the colonic motility. Consistently, rats injected with AAV-A53T excreted larger feces compared to rats injected with AAV-EV and littermate controls (Additional file 2: Fig. S1A), accompanied by a reduction in the fecal pellet count and total weight within 12 h (Fig. 1C, D). In addition, drying and weighing of the stools revealed that the fecal water content of the AAV-A53T-injected rats was much lower than that of the AAV-EV and controls (Fig. 1E). Furthermore, no significant differences were found in the assessments of gastrointestinal function between the control and AAV-EV groups (Fig. 1B–E). Nevertheless, motor behavior assessments at this time point revealed that rats in the AAV-A53T group did not exhibit motor deficits for which there were no significant differences in either the rotarod test or the open-field experiment (Additional file 2: Fig. S1B–E), while after 6 months of injection manifested reduced latency to fall, decreased total distance, as well as slower average velocity (Additional file 2: Fig. S1F–I).

Indeed, Luxol fast blue staining showed sparse vagal fibers and demyelination in AAV-A53T-injected rats (Fig. 1F–I), demonstrating that the vagus nerve was damaged. Strikingly, immunohistochemical staining of the medulla oblongata and the midbrain SNc of AAV-A53T-injected rats at the onset of gastrointestinal AutD revealed no signs of neuronal loss on the left side (Fig. 1J, K, R, N, O, S). After 4 months of injection, AAV-A53T-injected rats exhibited a significant reduction of cholinergic neurons in the left DMNV compared to the right (Fig. 1L, R), while at 6 months a marked loss of dopaminergic neurons in the left SNc was observed (Fig. 1Q, S). These findings were in line with autopsy studies by Braak et al., who proposed that α-synucleinopathy originates in the periphery while progressing along the vagus nerve to the DMNV, eventually involving the substantia nigra [22]. Thus, vagal injection of AAV-A53T results in prominent signs of AutD, preceding the onset of motor deficits and CNS neuronal abnormalities by at least 3 months, which can be utilized to generate prodromal PD models.

### Reduced intestinal blood flow and decreased nerve conduction velocity in rats with α-synucleinopathy in the vagus nerve

**Fig. 2:**
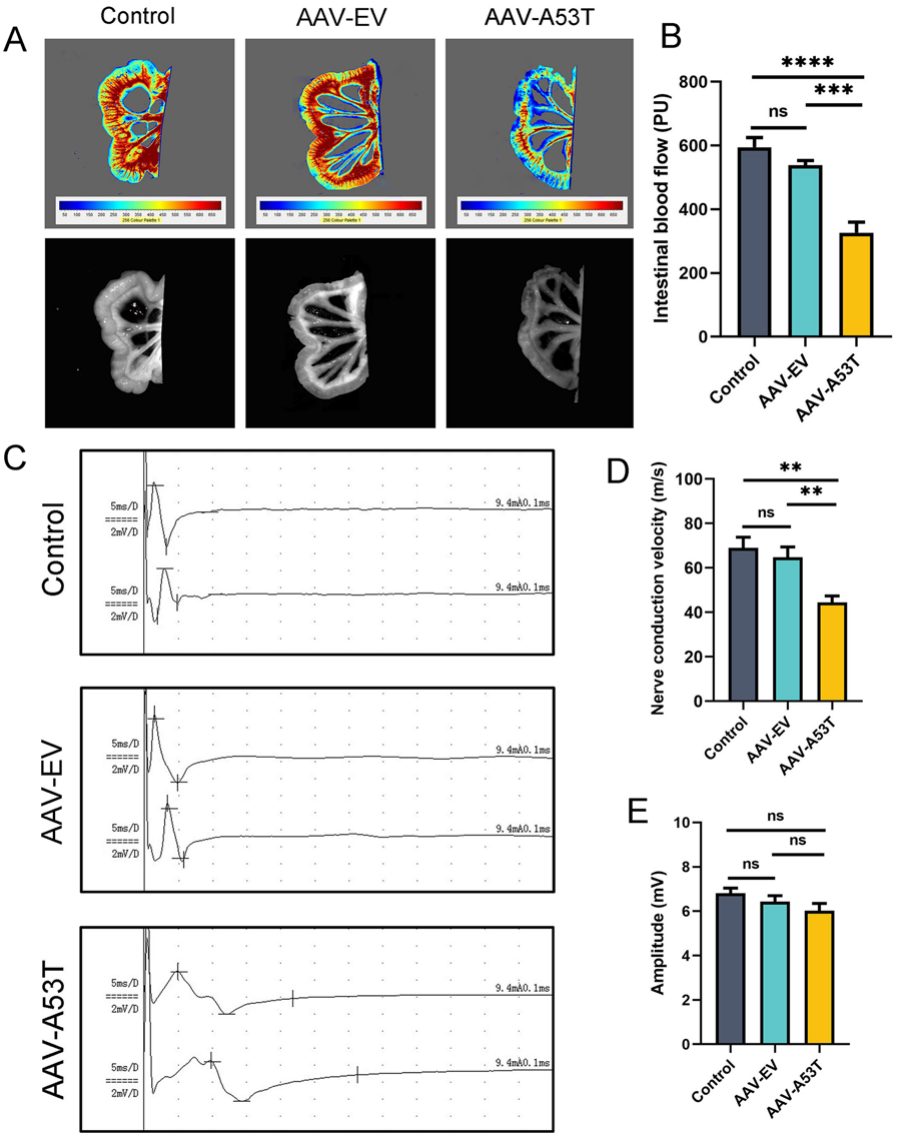
Vagal injection of AAV-A53T leads to reduced intestinal blood flow and decreased nerve conduction velocity. **A** Representative image of intestinal blood flow detected by laser Doppler at 3 months post-injection. **B** Average intestinal blood flow. **C** Representative images of the left vagus nerve compound action potential detected by the electromyography system at 3 months post-injection. **D** Average vagus nerve conduction velocity. **E** Average amplitude of vagus nerve compound action potential. *n* = 6 per group. Data were presented as mean ± SEM. Statistical significance was analyzed using one-way ANOVA followed by Bonferroni’s multiple comparison test. **P < 0.01, ***P < 0.001, ****P < 0.0001. ns, not significant

To further confirm vagal dysfunction, we examined the blood flow of the mesentery near the cecum segment in all rats at 3 months post-injection by laser Doppler flowmetry. As shown in Fig. 2A and B, the average blood flow was remarkably decreased in rats injected with AAV-A53T compared to rats injected with AAV-EV and controls, which may play a role in the development of gastrointestinal symptoms. Next, we performed a nerve conduction test with an electromyography system, a method for directly evaluating the function of vagus nerves (Additional file 2: Fig. S2). As illustrated in Fig. 2C, 3 months after injection, the vagus nerve compound action potential was presented with temporal dispersions in AAV-A53T-injected rats, accompanied by a slower nerve conduction velocity (Fig. 2D). Interestingly, the amplitude did not change significantly among groups (Fig. 2E), suggesting that the impaired vagus nerve activity may be primarily attributed to the myelin dysfunction. Moreover, there were no significant differences between the control group and the AAV-EV group in intestinal blood flow and nerve conduction velocity. Together, the reduced nerve conduction velocity induces a decline in intestinal blood flow, which is involved in gastrointestinal impairments and ultimately leads to constipation symptoms.

### Disrupted myelin sheaths and swollen SCs in the vagus nerve with α-synucleinopathy

**Fig. 3:**
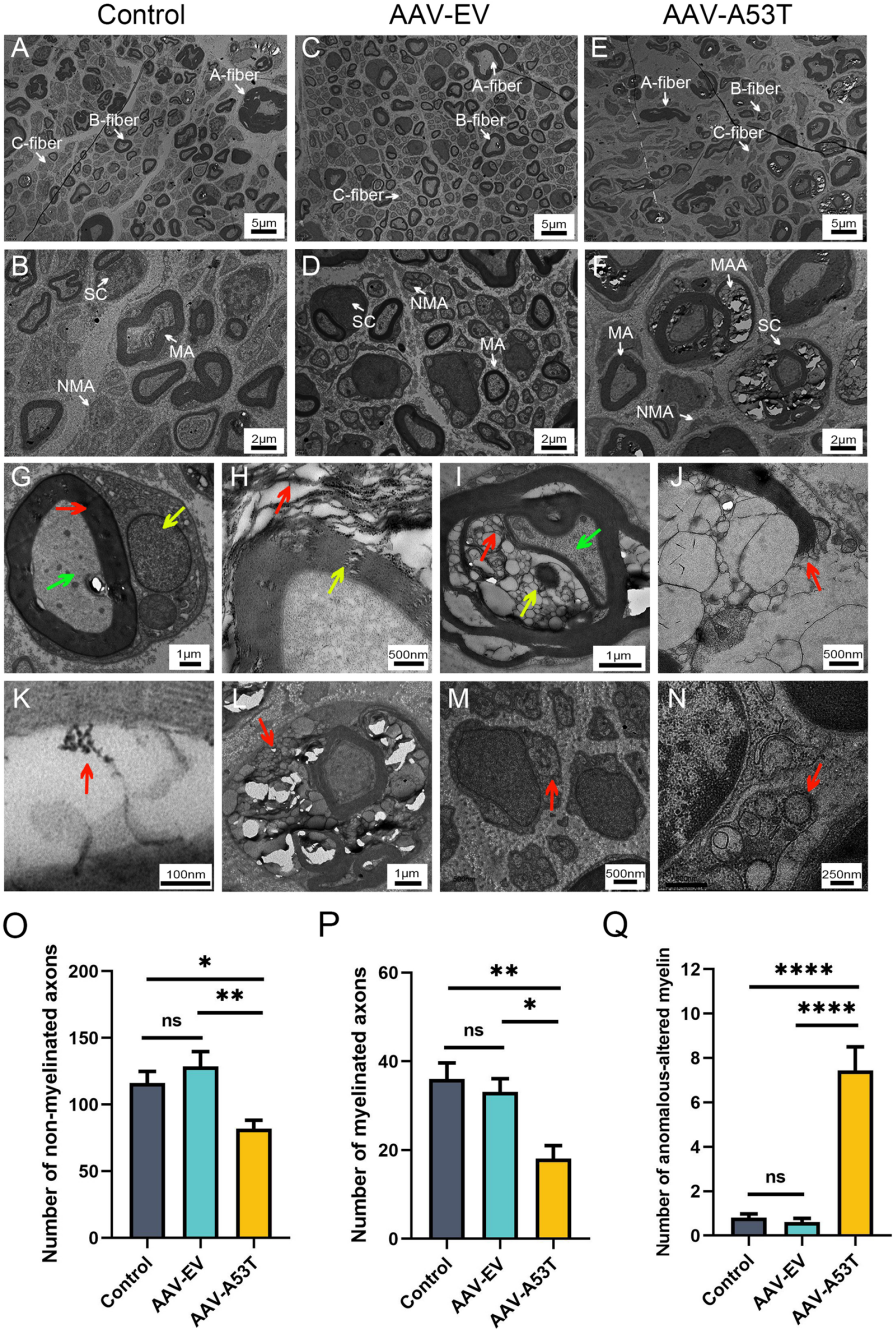
Disrupted myelin, abnormal alterations of SCs, and loss of axons are identified in AAV-A53T-injected rats. **A**–**F** Representative TEM images of the ultrastructure of the left vagus nerve among three groups at 3 months post-injection; Low magnification (**A**, **C**, **E**), scale bar = 5 μm; High magnification of the boxed regions in the top row (**B**, **D**, **F**), scale bar = 2 μm. **A**–**D** The structure of vagus nerves was not significantly different between the control and AAV-EV groups; Large myelinated A-fibers, medium diameter myelinated B-fibers, and small unmyelinated C-fibers were distributed uniformly, with round or oval SCs located at the periphery of the myelin sheath or wrapped around several unmyelinated axons and exhibited normal cellular morphology and structural integrity. **E**, **F** Abnormal alterations in myelin structure and the loss of B- and C-fibers. **G**–**N** Representative TEM images of the ultrastructure of myelin sheaths of left vagus nerves. **G** Normal myelin sheath structure in the control group (red arrow), axon (green arrow), and SC (yellow arrow), scale bar = 1 μm. **H**–**N** Left vagus nerve samples from AAV-A53T-injected rats. **H** Atrophic collapsed myelin sheath (red arrow) and sparse myelin sheath (yellow arrow), scale bar = 500 nm. **I** Lipid vacuoles in the delaminated myelin sheath (red arrow), squeezed axon (green arrow), and electron-dense areas of interest perhaps associated with the presumed α-synucleinopathy (yellow arrow), scale bar = 1 μm. **J** Fractured myelin sheath (red arrow), scale bar = 500 nm. **K** Abnormal protein aggregates deposited in the myelin gap (red arrow), scale bar = 100 nm. **L** Lipid vacuoles in swollen SCs (red arrow), scale bar = 1 μm. **M** Degenerated SCs (red arrow), scale bar = 500 nm. **N** Electron-dense areas of interest perhaps associated with the presumed α-synucleinopathy (red arrow), scale bar = 500 nm. Classification and counting numbers of non-myelinated axons (**O**), myelinated axons (**P**), and anomalous-altered myelin (**Q**) in left vagus nerve fibers. *n* = 6 per group. Data were presented as mean ± SEM. Statistical significance was analyzed using one-way ANOVA followed by Bonferroni’s multiple comparison test. *P < 0.05, **P < 0.01, ****P < 0.0001. MA, myelinated axons; NMA, non-myelinated axons; MAA, Myelin anomalous alteration

To specifically interrogate whether the myelin sheaths and axons are morphologically altered, TEM was conducted to evaluate the vagus nerve ultrastructure in rats at 3 months post-injection. As shown in Fig. 3A–D, the structure of vagus nerves was not markedly different between the control and AAV-EV groups. Large myelinated A-fibers, medium diameter myelinated B-fibers, and small unmyelinated C-fibers were distributed uniformly, along with round or oval SCs located at the periphery of the myelin sheath or wrapped around several unmyelinated axons, which exhibited normal cellular morphology and structural integrity. However, lesions in vagus nerves, including abnormal alterations of myelin structure and secondary axonal loss, were observed in AAV-A53T-injected rats, mainly involving B and C-fibers (Fig. 3E, F). As illustrated in Fig. 3G, the normal myelin sheath exhibits a dense lamellar structure resembling concentric circles in the TEM, whereas it is subjected to different types of pathological alterations in the vagus nerve with α-synucleinopathy. The myelin degenerated leading to delamination and collapse, while the morphology of the inner axon remained normal (Fig. 3H). Substantial lipid vacuoles were also found in the dense structure of the myelin and squeezed the axons, resulting in axonal deformation, with the deposition of electron-dense inclusions perhaps associated with the presumed α-synucleinopathy (Fig. 3I). More severely, there was a disruption of the myelin, with the broken sheath and its encircling axons being substituted by lipid vacuoles (Fig. 3J). Then, the myelin with structural disruption was locally magnified and depositions of abnormal protein aggregates were found in the myelin gap (Fig. 3K). These abnormalities may influence the physiological function of the myelin, resulting in impairments of the myelinated B-fibers that originate from the DMNV and are responsible for gastrointestinal motility. Moreover, we observed the changes in SCs via TEM and found that some SCs were swollen and filled with large amounts of lipid vacuoles (Fig. 3L). At the same time, some SCs degenerated resulting in the loss of pseudopods encircling nerve fibers (Fig. 3M), accompanied by the presence of electron-dense inclusions in the cytoplasm (Fig. 3N). Finally, the vagus nerve fibers were categorized and counted, and AAV-A53T-injected rats showed reduced numbers of myelinated and non-myelinated nerve fibers while increased abnormally altered myelin (Fig. 3O–Q), which may result in a decline in intestinal blood flow, slower nerve conduction velocity, as well as gastrointestinal symptoms. Consistent with the results in Figs. 1 and 2, the TEM results showed no significant differences between the control group and the AAV-EV group, suggesting that surgical operations and viral vectors were not sufficient to cause morphological and structural alterations of the vagus nerve myelin sheath and axons.

### p-α-Synuclein is deposited in SCs, but not in axons of the vagus nerve

**Fig. 4:**
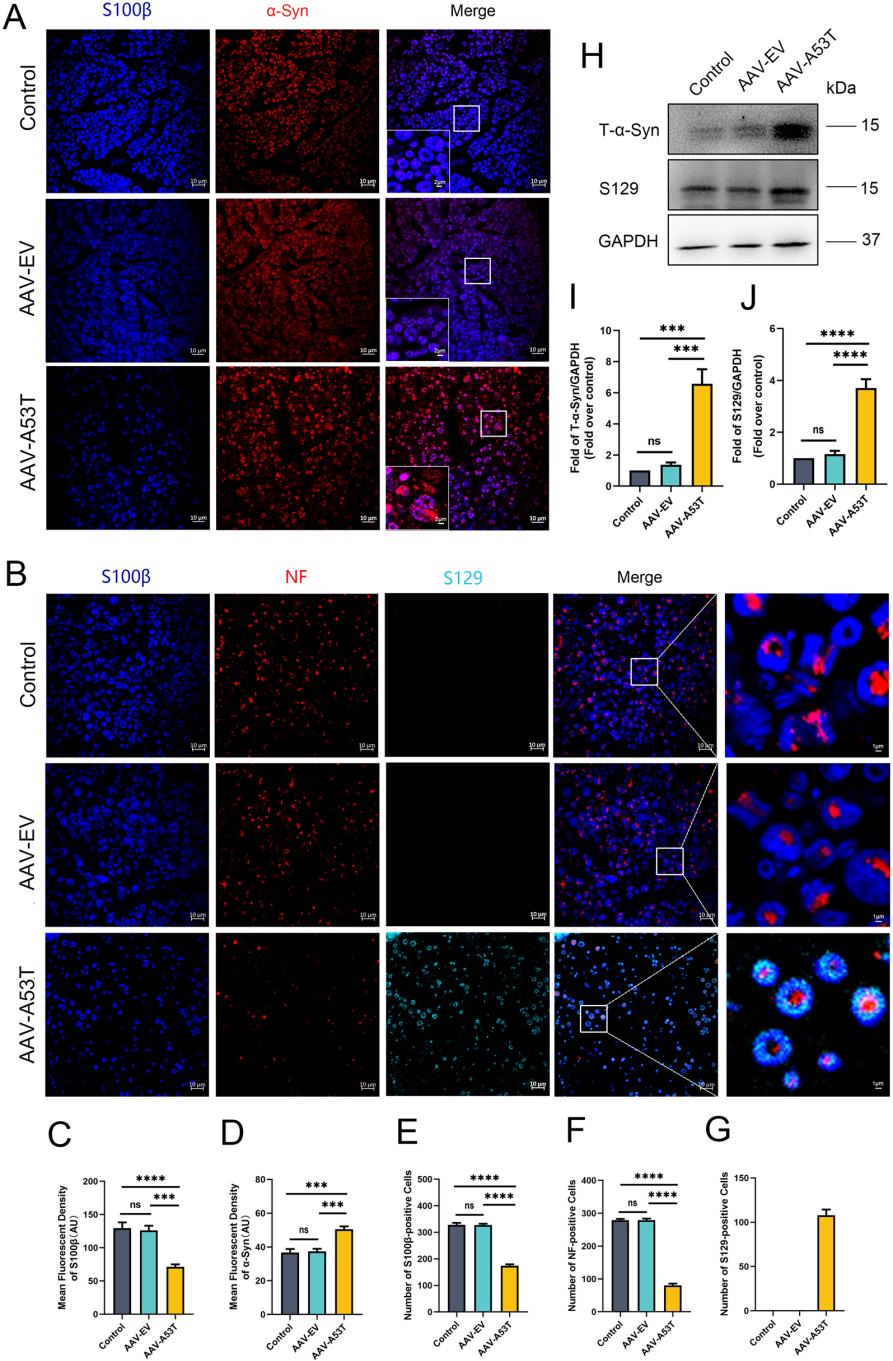
Vagal injection of AAV-A53T induces p-α-synuclein deposited in the SCs of vagus nerves. **A** Representative confocal image of S100β (blue) and α-synuclein (red) in the left vagus nerve at 3 months post-injection and quantitative analysis of the density (**C**, **D**). Scale bar = 10 μm. *n* = 6. **B** Representative confocal images of S100β (blue), NF (red), and S129 (light blue) in the left vagus nerve at 3 months post-injection and quantitative analysis of positive cells (**E**–**G**). Scale bar = 10 μm. *n* = 6. Representative western blot bands (**H**) and the statistical graph (**I**, **J**) of total α-synuclein and p-α-synuclein in the left vagus nerve. The protein levels were normalized to GAPDH and expressed as fold-over controls. *n* = 4. Data were presented as mean ± SEM. Statistical significance was analyzed using one-way ANOVA followed by Bonferroni’s multiple comparison test. ***P < 0.001, ****P < 0.0001. ns, not significant

In previous studies, we found that p-α-synuclein, a neuropathological hallmark of PD, was abnormally deposited in the SCs of the sural nerve of PD patients and the sciatic nerve of PD mice models [18–20]. We then asked whether p-α-synuclein is similarly deposited in the vagus nerve and involved in the pathogenesis of structural and functional defects. Immunofluorescent staining was performed on paraffin-embedded rat vagus nerve sections following 3 months of injection. Remarkably, total α-synuclein was elevated in AAV-A53T-injected rats versus AAV-EV-injected rats and controls, though not exclusively expressed in SCs (Fig. 4A, C, D). Furthermore, p-α-synuclein was markedly increased in AAV-A53T-injected rats and was barely observed in AAV-EV-injected rats and controls, which was positively correlated with the manifestation of vagal dysfunction (Fig. 4B, E–G). In line with this, western blotting of vagus nerve lysates revealed that both expressions of total α-synuclein and p-α-synuclein were escalated in AAV-A53T-injected rats (Fig. 4H–J). Next, we determined the localization of p-α-synuclein by co-staining with SCs-specific and axon-specific markers. Intriguingly, p-α-synuclein was deposited in SCs, but not in axons, suggesting that SCs deficits are responsible for vagal disturbances (Fig. 4B). In addition, aggregated α-synuclein was observed in the rat left vagus nerve and partially deposited on SCs (Additional file 2: Fig. S3). These results, together with TEM observations, further indicate that p-α-synuclein deposition within the SCs may be implicated in AutD in the prodromal phase of PD.

### p-α-Synuclein causes inflammatory responses via activating TLR2/MyD88/NF-κB signaling in rats and human SCs

**Fig. 5:**
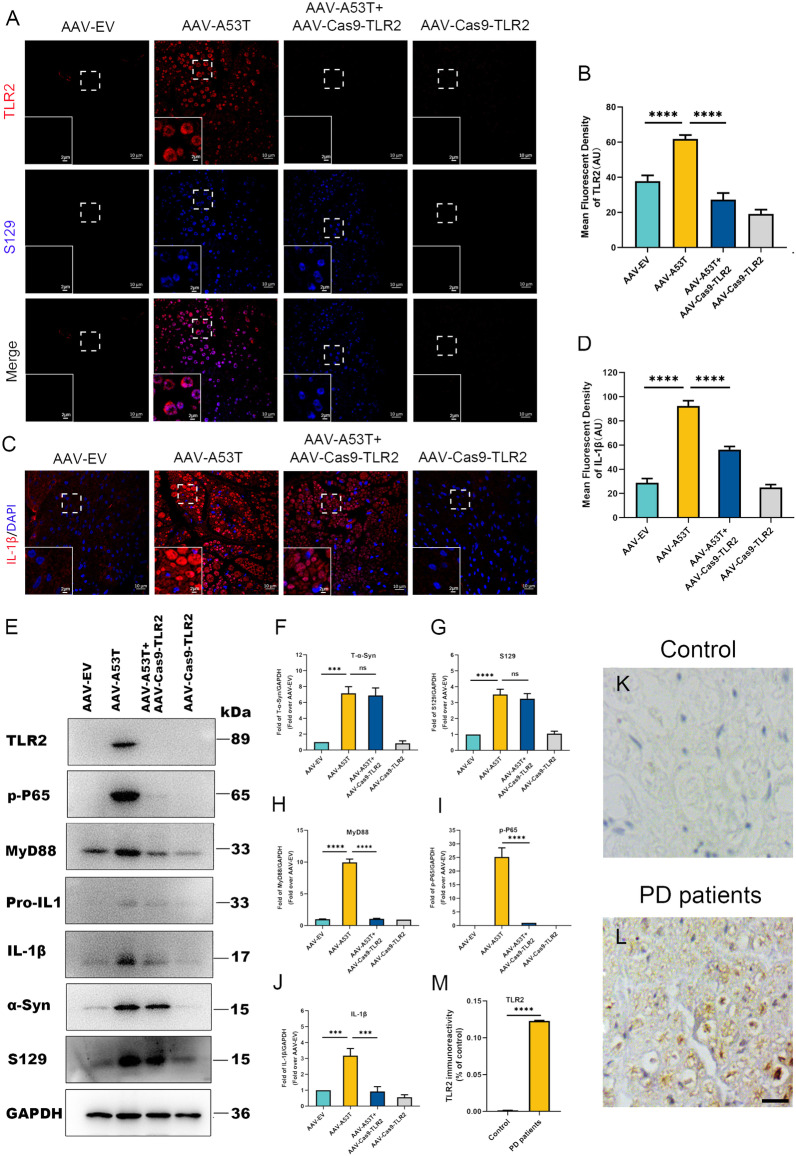
Deposition of p-α-synuclein in SCs induces inflammatory responses via activating TLR2/MyD88/NF-κB signaling. **A**, **B** Representative confocal images and quantitative analysis of TLR2 in the rat left vagus nerve at 3 months post-injection. Scale bar = 10 μm. *n* = 6. **C**, **D** Representative confocal images and quantitative analysis of IL-1β in the rat left vagus nerve at 3 months post-injection. Scale bar = 10 μm. *n* = 6. Representative western blot bands (**E**) and the statistical graph (**F**–**J**) of total α-synuclein, p-α-synuclein, MyD88, p-NF-κB p65, and IL-1β in the rat left vagus nerve at 3 months post-injection. The protein levels were normalized to GAPDH and expressed as fold over AAV-EV. *n* = 4. **K**–**M** Representative immunohistochemical staining and quantitative analysis of TLR2 in sural nerves of controls and PD patients. Scale bar = 10 μm. *n* = 6. Data were presented as mean ± SEM. Statistical significance was analyzed using two-way ANOVA followed by Bonferroni’s multiple comparison test in **B**, **D**, and **F**–**J**, and Student’s *t *test in **M**. ***P < 0.001, ****P < 0.0001. ns, not significant

Recent studies have shown that Toll-like receptors are involved in neuroinflammation in PD while SCs acting as peripheral nerve immune surveillance cells could upregulate TLR2 expression following activation [36, 37]. To clarify how the p-α-synuclein deposition contributes to inflammation in SCs and participates in the pathogenesis of vagal dysfunction, we performed immunofluorescence and found that TLR2 expression was markedly enhanced in rats injected with AAV-A53T compared to rats injected with AAV-EV (Fig. 5A, B). In line with this, the inflammatory cytokine IL-1β was higher in AAV-A53T-injected rats, in contrast to minimal expression in AAV-EV, indicating inflammatory reactions occur in vagal SCs (Fig. 5C, D). Furthermore, immunoblotting results showed that p-α-synuclein upregulated the expression of TLR2 in the vagus nerve, as well as MyD88, served as its adapter (Fig. 5E, H). NF-κB is a transcription factor of the TLR2-MyD88 signaling pathway, which is activated by phosphorylation. As shown in Fig. 5E, I, and J, p-NF-κB p65 was increased in the vagus nerve of AAV-A53T-injected rats, accompanied by a robust expression of IL-1β.

To further confirm the role of TLR2 signaling in the inflammation of the injured vagus nerve, we injected AAV-Cas9-TLR2 simultaneously in the vagus nerve of rats with α-synucleinopathy. Compared with rats injected with AAV-A53T, IL-1β intensity decreased markedly in the vagus nerve of rats injected with AAV-A53T and AAV-Cas9-TLR2 (Fig. 5C, D). Moreover, western blotting results identified that the expression of MyD88 and p-NF-κB p65 declined significantly with the depletion of TLR2, accompanied by reduced levels of inflammatory cytokine IL-1β (Fig. 5E, H–J). Hence, these findings indicate that vagal lesions are dependent on the activation of the TLR2 signaling pathway, which could be effectively attenuated by silencing TLR2. However, we did not find distinct differences in α-synuclein expression between these groups, either total α-synuclein or p-α-synuclein (Fig. 5E–G), implying that ablating TLR2 may not reverse α-synuclein pathology, but can alleviate local neuroinflammation.

Notably, our recent study observed that SCs of the sural nerve in PD patients were activated, with elevated levels of inflammatory cytokines including IL-1β, interleukin-6 (IL-6), and tumor necrosis factor-alpha compared to healthy controls [19]. We further performed immunohistochemical staining on the sural nerve of PD patients and found that the expression of TLR2 was increased (Fig. 5K–M). Together, these findings demonstrate that p-α-synuclein deposition in SCs induces activation of the TLR2/MyD88/NF-κB inflammatory pathway, which in turn participates in vagal nerve lesions.

### Depletion of TLR2 attenuates p-α-synuclein-induced AutD and vagus nerve demyelination and axonal loss

**Fig. 6:**
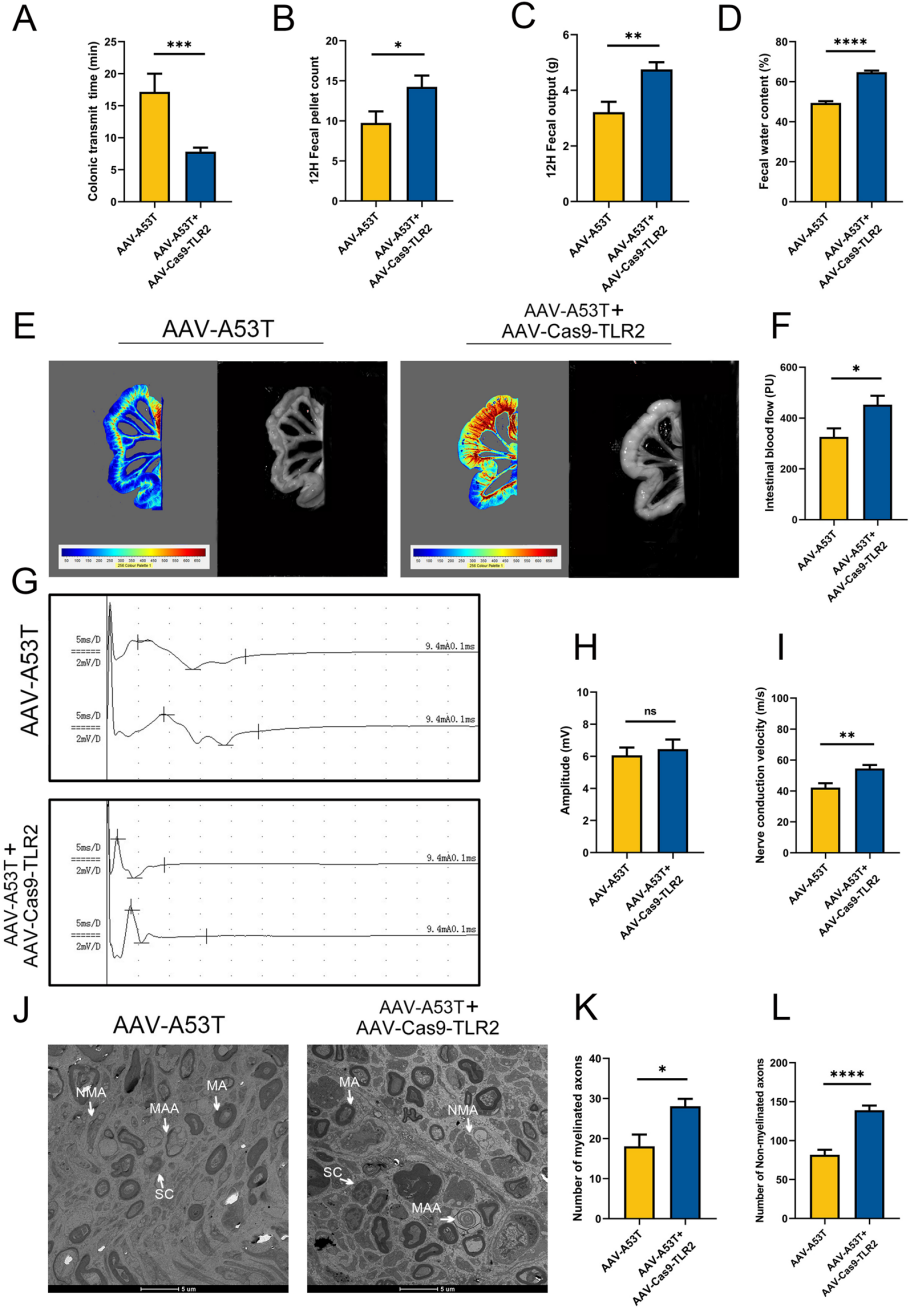
Silencing of TLR2 expression attenuates p-α-synuclein-induced AutD and vagus nerve lesions. SD rats were subjected to vagal injection with AAV-A53T or AAV-A53T as well as AAV-Cas9-TLR2 and then evaluated with gastrointestinal autonomic function 3 months later. **A** colonic transit time. **B** 12 h fecal pellet count and total weight (**C**). **D** 12 h fecal water content. **E** Representative images of intestinal blood flow detected by laser Doppler. **F** Average intestinal blood flow. **G** Representative images of the left vagus nerve conduction detected by the electromyography system. **H** Average vagus nerve conduction velocity. **I** Average amplitude of vagus nerve compound action potential. **J** Representative TEM images of the ultrastructure of the left vagus nerve in rats. Scale bar = 5 μm. Classification and counting numbers of non-myelinated axons (**K**) and myelinated axons (**L**) in left vagus nerve fibers. **A**–**D**: *n* = 12 per group; **E**–**L**: *n* = 6 per group. Data were presented as mean ± SEM. Statistical significance was analyzed using Student’s *t *test. *P < 0.05, **P < 0.01, ***P < 0.001, ****P < 0.0001. ns, not significant; MA, myelinated axons; NMA, non-myelinated axons; MAA, Myelin anomalous alteration

To investigate whether TLR2 signaling is critical for the development of AutD, we assessed alterations in gastrointestinal symptoms, vagal function and ultrastructure in rats 3 months after TLR2 knockdown. Rats injected with AAV-A53T and AAV-Cas9-TLR2 exhibited shortened colonic transit time, increased fecal pellet counts and total weight within 12 h, and higher fecal water content, suggesting that blocking TLR2 signaling protects rats against gastrointestinal dysfunction (Fig. 6A–D). Consistently, as shown in Fig. 6E and F, intestinal blood flow was markedly increased, along with nerve conduction velocity was accelerated in rats injected with AAV-A53T and AAV-Cas9-TLR2 (Fig. 6G–I), indicating that silencing TLR2 expression attenuates vagal dysfunction. Strikingly, TEM analysis of myelin sheaths and axons in the vagus nerve found that TLR2 depletion alleviated damage in nervous tissue as evidenced by the diminished loss of myelinated and non-myelinated axons (Fig. 6J–L).

In addition, the results of the comparison between the AAV-A53T and AAV-Cas9-TLR2, and the control group showed that the TLR2 knockdown did not completely reverse the vagus nerve dysfunction induced by α-synuclein, suggesting that the TLR2 inflammatory pathway may be one of the pathological mechanisms underlying AutD in the prodromal PD (Additional file 2: Fig. S5).

## Discussions

By the time PD could be diagnosed according to its cardinal motor manifestations, neurodegeneration was not only in the substantia nigra but more broadly, which has evolved over many years [38, 39]. Evidence from prospective research continues to confirm the concept of prodromal PD and has contributed to the development of PD prediction models [40–42]. Of note, the autonomic nervous system seems to be a hopeful target for assaying individuals in the prodromal phase and monitoring the therapeutic effects of interventions in the future [8]. Here, we described a rat model of prodromal PD, performed by injecting AAV-A53T into the vagus nerve, that exhibited AutD as determined by behavioral, functional, and pathological examinations. Our study revealed for the first time that ultrastructural lesions of the vagal SCs were present in the prodromal PD model, which could lead to decreased nerve conduction velocity and reduced intestinal blood flow, and this was directly involved in the development of AutD. Moreover, our data demonstrated that p-α-synuclein is deposited in SCs of the vagus nerve but not in axons while being associated with higher expression of TLR2. In addition, MyD88 and its downstream transcription factor NF-κB p65, as well as the inflammatory cytokines IL-1β, were upregulated in the damaged vagus nerve, triggering focal neuroinflammation, which subsequently led to the destruction of neural integrity. Furthermore, silencing the TLR2 gene not only reduced inflammatory cytokine expression but also ameliorated demyelination and secondary axonal loss in vagus nerves, consequently improving autonomic function in rats.

AutD, a crucial feature of prodromal PD, afflicts approximately 90% of patients with PD and correlates with faster disease progression and shorter survival [9, 43], yet it remains inconclusive regarding its pathological mechanisms, in part due to the lack of appropriate animal models to mimic the chronic and progressive nature of the pathological stages of PD. In this study, a valuable model for prodromal PD was performed by selectively inducing α-synucleinopathy in the vagus nerve, which can exhibit AutD preceding motor deficits and CNS lesions. Consistently, Lucia et al. found that the A53T α-synuclein transgenic mice display gastrointestinal dysfunction months before the motor abnormalities and α-synuclein pathology in the CNS, attributing to the accumulation of α-synuclein in the enteric neurons system [31]. Findings from Collin et al. suggested that injection into the duodenal wall with α-synuclein preformed fibers in adult mice promoted a local inflammatory and disrupted enteric neuron system connectivity [44]. A study published in 2021 showed that α-synuclein contributes to Kv4 channelopathy in vagal motoneurons of a mouse model of prodromal PD, which in turn reduces gastrointestinal motility [30]. The main distinguishing factors between these investigations and ours were different forms of α-synuclein, injection locations, as well as time points observed. We utilized the human mutant A53T α-synuclein, which is a confirmed pathogenic gene for familial PD [45, 46]. Indeed, the association between the impaired intestinal epithelial barrier induced by the enteric α-synuclein accumulation and the bowel symptoms has also been reported [47], although results from gastrointestinal tract permeability research were highly heterogeneous [48, 49]. These observations, together with our results, demonstrate that the pathogenesis of gastrointestinal dysfunction in PD is complicated, involving the neural pathways of the gut, the autonomic nervous system, and the DMNV. It should be emphasized that by using this model, the present study focused on investigating the underlying mechanisms of AutD in the prodromal phase of PD.

Furthermore, after 4 months of vagal injection, the typical central neuropathology and motor symptoms of PD developed in a chronological and regional sequence in this model corresponding to the Braak staging system [22, 50, 51]. Consistently, Rusconi et al. found that injecting hα-synuclein-carrying AAVs into the rat left vagus nerve could induce a progressive neuronal loss in the locus coeruleus, accompanied by pronounced microglial activation and astrocytosis [52]. Recently, Lindberg et al. reported vagal injection of human α-synuclein‐encoding AAVs in mice could lead to the trans‐synaptic spread of α-synuclein through the brain and the loss of dopaminergic neurons [53].

The vagus nerve originates from the medulla oblongata and, as the main component of the parasympathetic nervous system, regulates functions of the stomach, small intestine, and colon up to the splenic flexure within the abdominal cavity. In fact, the vagus nerve is a mixed cranial nerve containing motor efferent fibers and sensory afferent fibers of the somatic and special viscera, which can also be categorized as large myelinated A-fibers, medium diameter myelinated B-fibers, and small unmyelinated C-fibers according to their physical and electrical conductance properties [54]. In our study, observations from TEM confirmed that the ultrastructure of vagus nerves was abnormally altered, with lesions primarily affecting B- and C-fibers, which are responsible for visceral motor and sensory signaling, and this may provide direct evidence for the occurrence of gastrointestinal AutD in prodromal PD. Moreover, our study clarified the presence of vagus nerve myelin and axonal damage in the AutD prodromal PD model, which could induce retrograde axonal degeneration, leading to neuronal death in the DMNV and possibly spreading to other vulnerable brain regions. Previous clinical studies evaluating the cross-sectional areas of vagus nerves using ultrasonography, found evidence of smaller vagus nerves in patients with PD compared to healthy controls, reflecting vagal neuronal loss due to α-synuclein aggregates [55–57], which has been confirmed by a postmortem histopathological study analyzing the distribution of α-synucleinopathy in the brain and peripheral autonomic nervous system [58]. Indeed, epidemiological studies have found an association between vagotomy and the subsequent decreased risk of developing PD [23, 24]. Overall, these results shed light on the potential role of the vagus nerve in the AutD pathogenesis of PD.

To date, multiple studies have reported that autonomic innervations of the skin, heart, and gut are affected by α-synucleinopathy in the early stage of PD and might initiate α-synuclein spread to the CNS through the autonomic connectome [8, 59, 60]. Our subsequent work further demonstrated that p-α-synuclein was deposited in SCs, both in the sural nerve of PD patients and the sciatic nerve of animal models [18–20]. Notably, in the peripheral nervous system, SCs have been shown to play an essential role in the secretion of neurotrophic factors and the regulation of immune responses [37, 61]. In the current study, p-α-synuclein was shown for the first time to selectively deposit in SCs of the vagus nerve, which may be the primary pathological basis for vagal dysfunction. Moreover, the accumulating body of evidence demonstrates that TLR2, acting as a receptor for pathological α-synuclein, can mediate neuroinflammation in PD and contribute to neurodegeneration, eventually resulting in disease progression [36, 62–64]. Findings from an in vitro study have shown that blocking the TLR2 signaling pathway could inhibit neuroinflammation and ameliorate disease pathology in the CNS of PD [65]. Dutta et al. found that the TLR2/MyD88/NF-κB pathway plays an important role in the spread of α-synuclein, and targeting this pathway via peptides or genetic deletion of TLR2 could reduce α-synuclein spreading and protect dopaminergic neurons [66]. Consistently, the deposition of p-α-synuclein was found to induce vagus nerve dysfunction and structural damage through a TLR2-mediated inflammatory response in our study. However, it is worth pointing out that deletion of TLR2 did not diminish the deposition of p-α-synuclein, indicating that the process by which α-synuclein was phosphorylated occurs at least partially upstream of TLR2 signaling and that a single anti-inflammatory therapy may not address a substantial amount of underlying pathology or prevent the development of PD [67]. The vulnerability of myenteric neurons to α-synuclein pathology, as well as the possible interaction of propagated α-synuclein with the deleterious catecholaminergic profile and together enhance neuronal damage, are also suggested to be associated with the AutD in prodromal PD [8].

Given that constipation is one of the most common AutD in prodromal PD and is well established to be associated with an increased risk of developing PD, we focused on gastrointestinal symptoms in this work, whereas further investigations are needed for cardiovascular impairment, urinary abnormalities, and sexual dysfunction. Additionally, in this study, the findings in the control and AAV-EV groups were indeed different, yet not statistically significant, indicating surgical operations and viral vectors may cause vagus nerve changes but were not sufficient to induce vagal dysfunction and structural damage. Moreover, AutD is not only observed in patients with PD but also in those with other neurodegeneration disorders, such as pure autonomic failure and dementia with Lewy bodies, suggesting that the peripheral autonomic nervous system can be considered as an additional potential modulator of disease onset and progression while requiring long-term monitoring.

## Conclusions

In summary, our novel observations suggest that overexpression of human mutated A53T α-synuclein in the vagus nerve could induce abnormal deposition of p-α-synuclein in SCs, initiating local neuroinflammation through activating the TLR2/MyD88/NF-κB signaling pathway, eliciting ultrastructural lesions in SCs and replicating the AutD characteristics of prodromal PD. Together, our study provides an opportunity for a deeper understanding of the pathological mechanisms underlying prodromal symptoms of PD and may contribute to the emergence of effective therapies to delay or prevent the progression of PD from the peripheral autonomic nervous system to the CNS.

## Supplementary Information

**Additional file 1.** Movie recording of the excretion of steel bead in an AAV-A53T-injected rat at 3 months post-injection.

**Additional file 2: Figure S1.** Vagal injection of AAV-A53T induces gastrointestinal dysfunction and subsequent motor deficits. **A** Representative image of stools collected in 12 h from rats among three groups at 3 months post-injection. Note that fecal pellets from AAV-A53T-injected rats are longer but less abundant. Latency to fall in the rotarod test (**B**), total distance (**C**) and average velocity (**D**), and representative images of the behavioral trajectories of rats in the open field test (**E**) at 3 months post-injection. Latency to fall in the rotarod test (**F**), total distance (**G**) and average velocity (**H**), and representative images of the behavioral trajectories of rats in the open field test (**I**) at 6 months post-injection. *n* = 12 per group. Data were presented as mean ± SEM. Statistical significance was analyzed using one-way ANOVA followed by Bonferroni’s multiple comparison test. ****P < 0.0001. ns, not significant. **Figure S2.** Schematic diagram of electrophysiological testing of the vagus nerve. ①Vagus nerve; ② Stimulation electrode; ③ Recording electrode; ④ Recording electrode; ⑤ Ground electrode; ⑥ Ground electrode. **Figure S3.** Vagal injection of AAV-A53T induces aggregated α-synuclein deposited in the SCs of vagus nerves. **A** Representative confocal images of S100β (green), NF (violet), and MJF-14 (red) in the left vagus nerve at 3 months post-injection. Scale bar = 10 μm. **B** Representative confocal images of S129 (blue) and MJF-14 (red) in the left vagus nerve at 3 months post-injection. Scale bar = 10 μm. **Figure S4.** Vagal injection of AAV- Cas9-TLR2 induces a decline in TLR2 expression. Representative western blot bands (**A**) and statistical graph (**B**) of TLR2 in vagus nerves in rats among three groups at 3 months post-injection. The protein levels were normalized to GAPDH and expressed as fold-over control. *n* = 4. Data were presented as mean ± SEM. Statistical significance was analyzed using one-way ANOVA followed by Bonferroni’s multiple comparison test. ***P < 0.001, ****P < 0.0001. ns, not significant. **Figure S5.** TLR2 knockdown could partially reverse vagus nerve dysfunction. **A** colonic transit time. **B** 12 h fecal pellet count. **C** 12 h fecal total weight. **D** 12 h fecal water content. **E** Average intestinal blood flow. **F** Average vagus nerve conduction velocity. **G** Average amplitude of vagus nerve compound action potential. Counting numbers of non-myelinated axons (**H**) and myelinated axons (**I**) in the left vagus nerve at 3 months post-injection. **A**–**D**: *n* = 12 per group; **E**–**I**: *n* = 6 per group. Data were presented as mean ± SEM. Statistical significance was analyzed using Student’s *t *test. *P < 0.05, ****P < 0.0001, ns = not significant.

## Data Availability

The datasets used and/or analysed during the current study are available from the corresponding author upon reasonable request.

## Abbreviations

PD: Parkinson’s disease
AutD: Autonomic dysfunction
SCs: Schwann cells
CNS: Central nervous system
DMNV: Dorsal motor nucleus of the vagus
AAVs: Adeno-associated viruses
TLR2: Toll-like receptor 2
TEM: Transmission electron microscopy
IL-1β: Interleukin-1 beta
IL-6: Interleukin-6
ChAT: Choline acetyltransferase
TH: Tyrosine hydroxylase
NST: Nucleus of solitary tract
CC: Central canal
L/RDMX: Left/right dorsal motor nucleus of vagus nerve
L/RSNc: Left/right substantia nigra pars compacta
VTA: Ventral tegmental area
SNr: Substantia nigra pars reticulata
MA: Myelinated axons
NMA: Non-myelinated axons
MAA: Myelin anomalous alteration
