# Supplementary material for: α-Synuclein induces prodromal symptoms of Parkinson’s disease via activating TLR2/MyD88/NF-κB pathway in Schwann cells of vagus nerve in a rat model
Source: J Neuroinflammation. 2023 Feb 14;20:36. doi: 10.1186/s12974-023-02720-1 (PMC9926693; doi:10.1186/s12974-023-02720-1)
Supplement: Supplementary file 2 — Additional file 2: Figure S1. Vagal injection of AAV-A53T induces gastrointestinal dysfunction and subsequent motor deficits. A Representative image of stools collected in 12 h from rats among three groups at 3 months post-injection. Note that fecal pellets from AAV-A53T-injected rats are longer but less abundant. Latency to fall in the rotarod test (B), total distance (C) and average velocity (D), and representative images of the behavioral trajectories of rats in the open field test (E) at 3 months post-injection. Latency to fall in the rotarod test (F), total distance (G) and average velocity (H), and representative images of the behavioral trajectories of rats in the open field test (I) at 6 months post-injection. n = 12 per group. Data were presented as mean ± SEM. Statistical significance was analyzed using one-way ANOVA followed by Bonferroni’s multiple comparison test. ****P < 0.0001. ns, not significant. Figure S2. Schematic diagram of electrophysiological testing of the vagus nerve. ①Vagus nerve; ② Stimulation electrode; ③ Recording electrode; ④ Recording electrode; ⑤ Ground electrode; ⑥ Ground electrode. Figure S3. Vagal injection of AAV-A53T induces aggregated α-synuclein deposited in the SCs of vagus nerves. A Representative confocal images of S100β (green), NF (violet), and MJF-14 (red) in the left vagus nerve at 3 months post-injection. Scale bar = 10 μm. B Representative confocal images of S129 (blue) and MJF-14 (red) in the left vagus nerve at 3 months post-injection. Scale bar = 10 μm. Figure S4. Vagal injection of AAV- Cas9-TLR2 induces a decline in TLR2 expression. Representative western blot bands (A) and statistical graph (B) of TLR2 in vagus nerves in rats among three groups at 3 months post-injection. The protein levels were normalized to GAPDH and expressed as fold-over control. n = 4. Data were presented as mean ± SEM. Statistical significance was analyzed using one-way ANOVA followed by Bonferroni’s multiple comparison test. ***P < 0.001, [file 12974_2023_2720_MOESM2_ESM.docx]

**
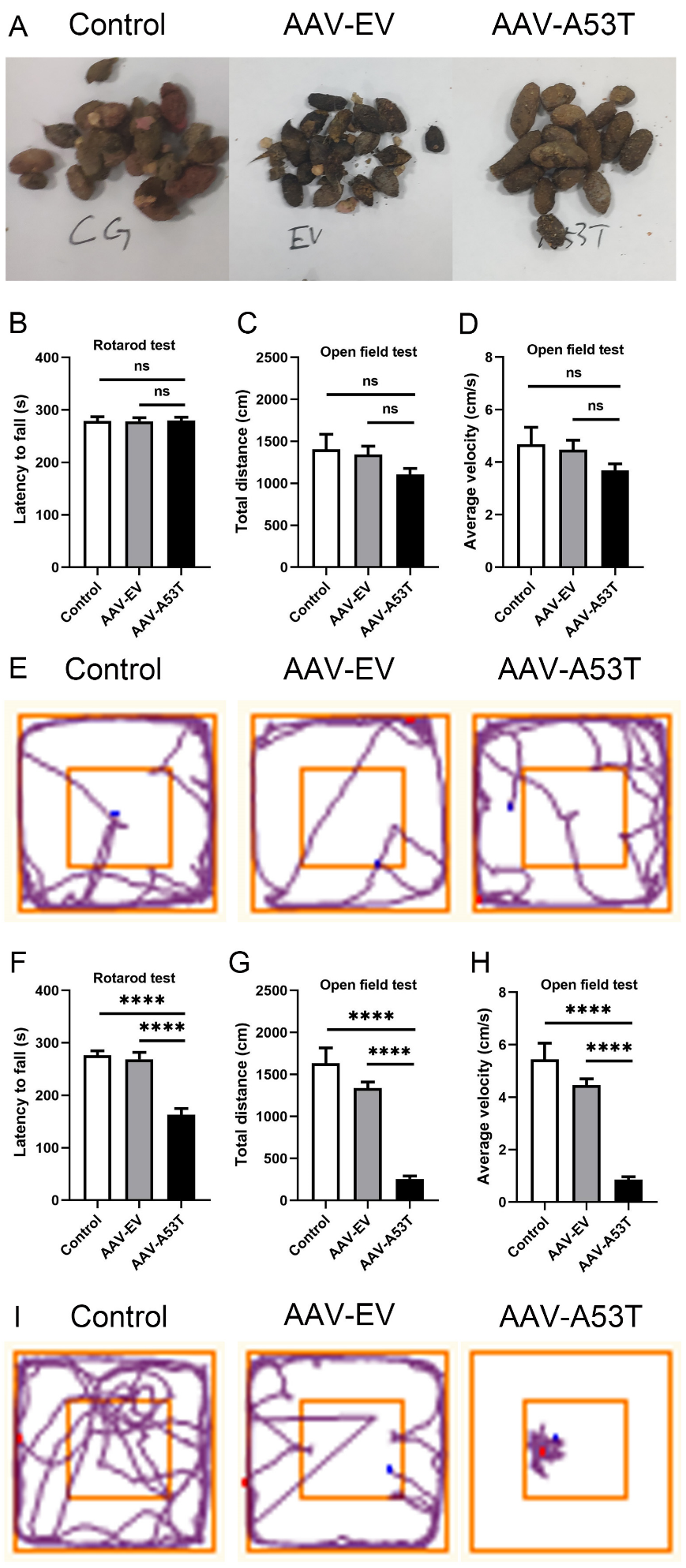
**

**Figure S1.** Vagal injection of AAV-A53T induces gastrointestinal dysfunction and subsequent motor deficits. (A) Representative image of stools collected in 12 hours from rats among three groups at 3 months post-injection. Note that fecal pellets from AAV-A53T-injected rats are longer but less abundant. Latency to fall in the rotarod test (B), total distance (C) and average velocity (D), and representative images of the behavioral trajectories of rats in the open field test (E) at 3 months post-injection. Latency to fall in the rotarod test (F), total distance (G) and average velocity (H), and representative images of the behavioral trajectories of rats in the open field test (I) at 6 months post-injection. n = 12 per group. Data were presented as mean ± SEM. Statistical significance was analyzed using one-way ANOVA followed by Bonferroni’s multiple comparison test. *****P* < 0.0001. ns = not significant


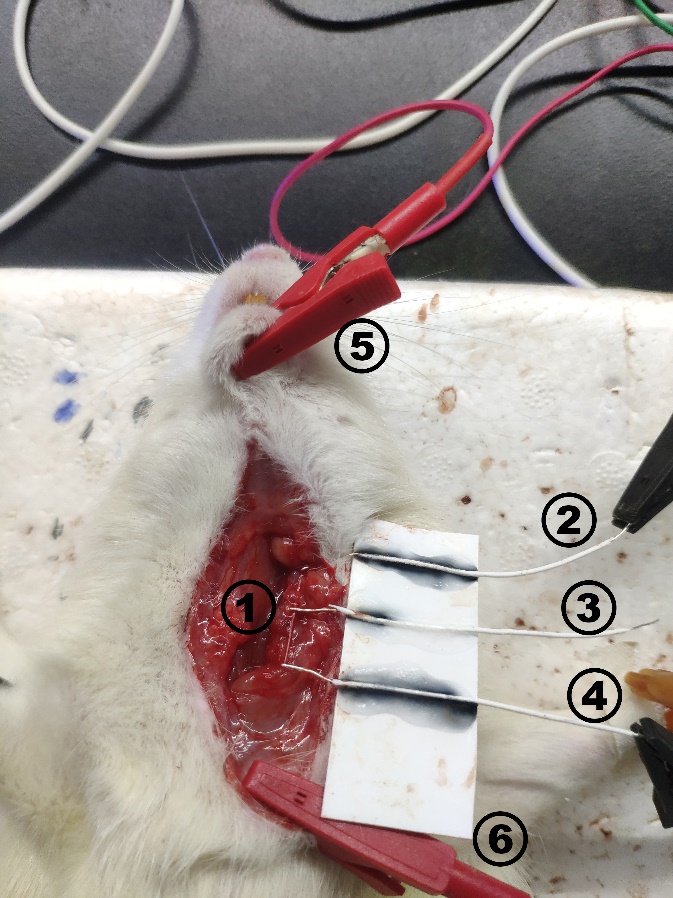


**Figure S2.** Schematic diagram of electrophysiological testing of the vagus nerve. ①Vagus nerve; ② Stimulation electrode; ③ Recording electrode; ④ Recording electrode; ⑤ Ground electrode; ⑥ Ground electrode


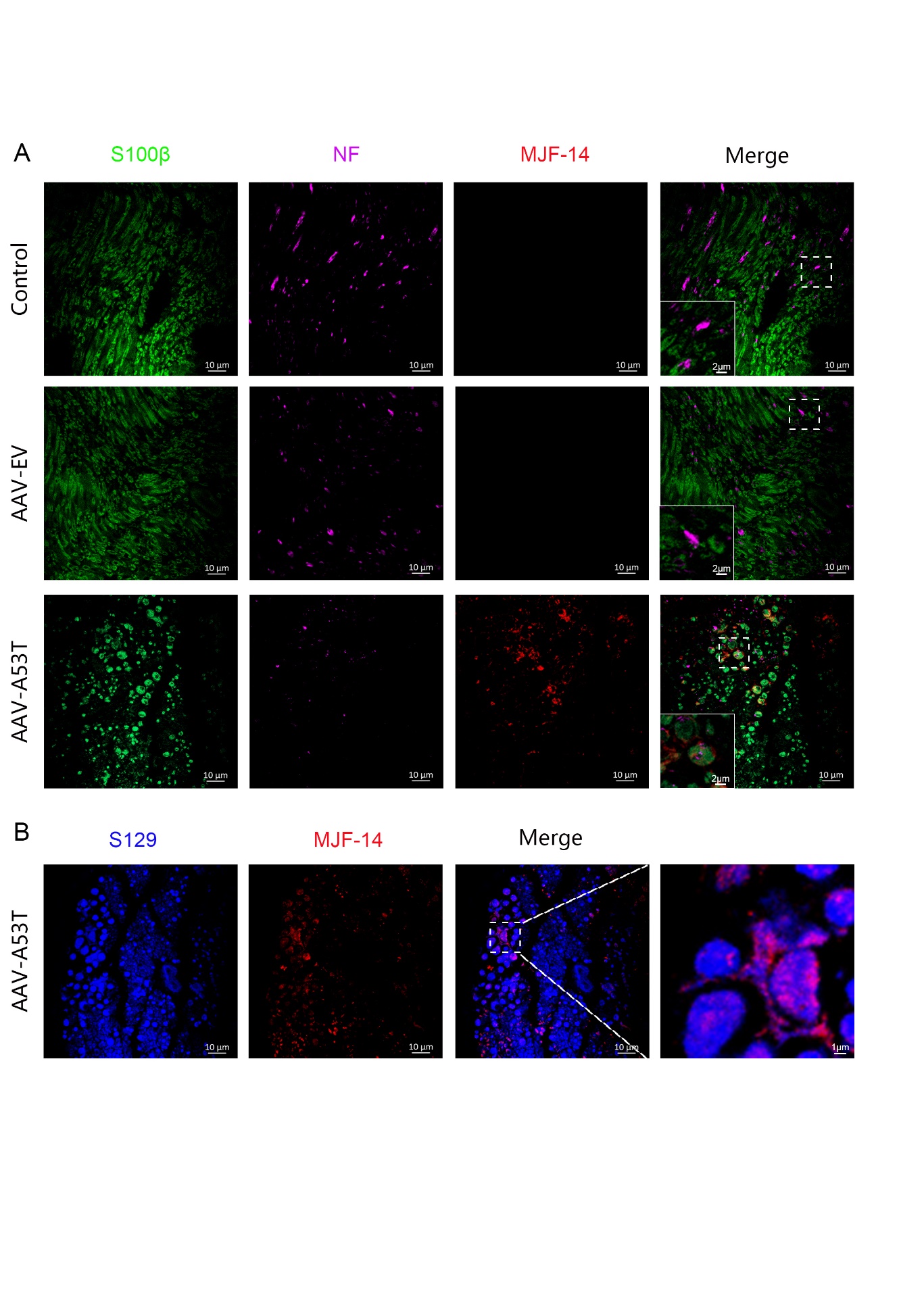


**Figure S3.** Vagal injection of AAV-A53T induces aggregated α-synuclein deposited in the SCs of vagus nerves. (A) Representative confocal images of S100β (green), NF (violet), and MJF-14 (red) in the left vagus nerve at 3 months post-injection. Scale bar = 10 μm. (B) Representative confocal images of S129 (blue) and MJF-14 (red) in the left vagus nerve at 3 months post-injection. Scale bar = 10 μm.


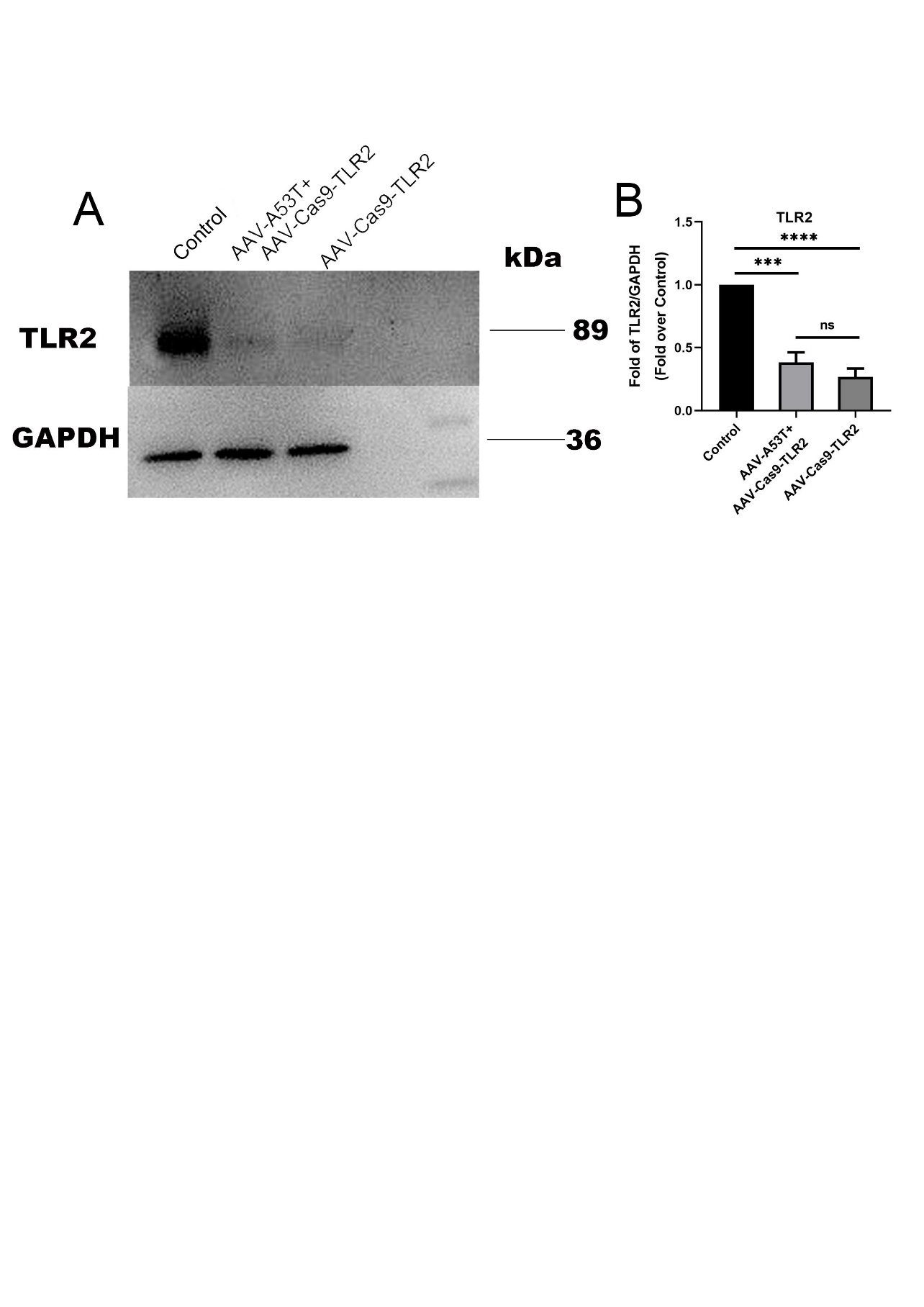


**Figure S4.** Vagal injection of AAV- Cas9-TLR2 induces a decline in TLR2 expression. Representative western blot bands (A) and statistical graph (B) of TLR2 in vagus nerves in rats among three groups at 3 months post-injection. The protein levels were normalized to GAPDH and expressed as fold over control. n = 4. Data were presented as mean ± SEM. Statistical significance was analyzed using one-way ANOVA followed by Bonferroni’s multiple comparison test. ****P* < 0.001, *****P* < 0.0001. ns = not significant


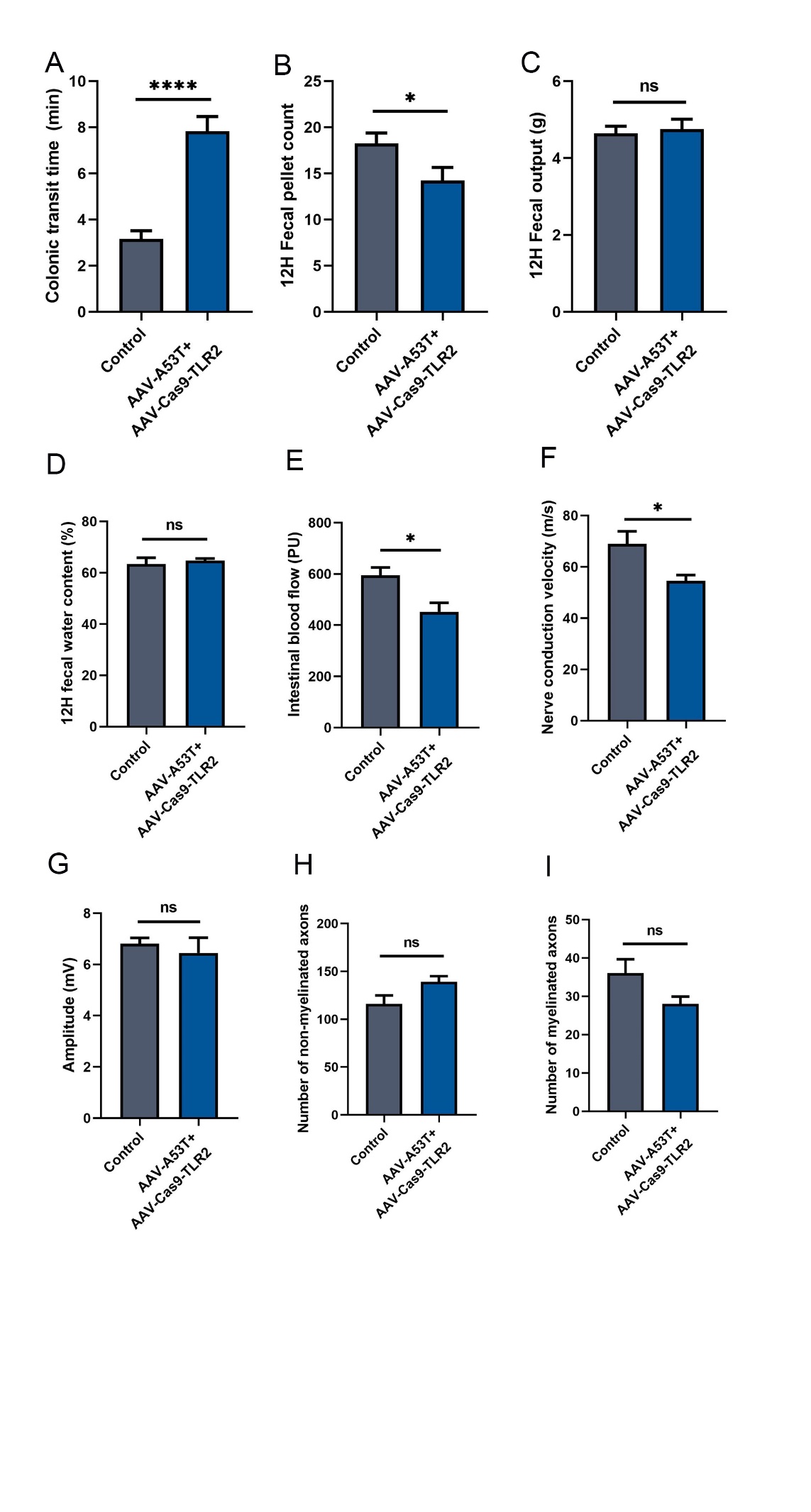


**Figure S5.** TLR2 knockdown could partially reverse vagus nerve dysfunction. (A) colonic transit time. (B) 12 hours fecal pellet count. (C) 12 hours fecal total weight. (D) 12 hours fecal water content. (E) Average intestinal blood flow. (F) Average vagus nerve conduction velocity. (G) Average amplitude of vagus nerve compound action potential. Counting numbers of non-myelinated axons (H) and myelinated axons (I) in the left vagus nerve at 3 months post-injection. A-D: n = 12 per group; E-I: n = 6 per group. Data were presented as mean ± SEM. Statistical significance was analyzed using Student’s t-test. **P* < 0.05, *****P* < 0.0001, ns = not significant
